# Effects of exercise or metformin on myokine concentrations in patients with breast and colorectal cancer: A phase II multi‐centre factorial randomized trial

**DOI:** 10.1002/jcsm.13509

**Published:** 2024-06-18

**Authors:** Justin C. Brown, Guillaume Spielmann, Shengping Yang, Stephanie L. E. Compton, Lee W. Jones, Melinda L. Irwin, Jennifer A. Ligibel, Jeffrey A. Meyerhardt

**Affiliations:** ^1^ Pennington Biomedical Research Center Baton Rouge LA USA; ^2^ LSU Health Sciences Center New Orleans School of Medicine New Orleans LA USA; ^3^ Stanley S. Scott Cancer Center Louisiana State University Health Sciences Center New Orleans LA USA; ^4^ Louisiana State University Baton Rouge LA USA; ^5^ Memorial Sloan Kettering Cancer Center New York NY USA; ^6^ Yale University New Haven CT USA; ^7^ Dana‐Farber Cancer Institute Boston MA USA

**Keywords:** biomarkers, diabetes, muscle, physical activity, recurrence, survival

## Abstract

**Background:**

Physical activity and metformin pharmacotherapy are associated with improved clinical outcomes in breast and colorectal cancer survivors. Myokines are cytokines secreted from skeletal muscle that may mediate these associations.

**Methods:**

This hypothesis‐generating analysis used biospecimens collected from a multi‐centre 2 × 2 factorial randomized design of 116 patients with stage I–III breast and colorectal cancer who were randomized to 12 weeks of (1) aerobic exercise (moderate intensity titrated to 220 min/week); (2) metformin (850 mg daily for 2 weeks and then titrated to 850 mg twice per day); (3) aerobic exercise and metformin; or (4) control. Fourteen myokines were quantified using a multiplex panel. Myokine concentrations were log‐transformed, and main effects analyses were conducted using linear mixed‐effects regression models. The type I error rate was controlled with the Holm sequential testing procedure.

**Results:**

Randomization to exercise increased leukaemia inhibitory factor (1.26 pg/mL, 95% confidence interval [CI]: 0.69, 1.84; adjusted *P* = 0.001) and interleukin‐15 (2.23 pg/mL, 95% CI: 0.87, 3.60; adjusted *P* = 0.013) compared with randomization to no exercise. Randomization to metformin decreased apelin (−2.69 pg/mL, 95% CI: −4.31, −1.07; adjusted *P* = 0.014) and interleukin‐15 (−1.74 pg/mL, 95% CI: −2.79, −0.69; adjusted *P* = 0.013) compared with randomization to no metformin. Metformin decreased myostatin, irisin, oncostatin M, fibroblast growth factor 21 and osteocrin; however, these changes were not statistically significant after correction for multiple comparisons.

**Conclusions:**

This pilot study demonstrates that randomization to exercise and metformin elicit unique effects on myokine concentrations in cancer patients. This hypothesis‐generating observation warrants further basic, translational and clinical investigation and replication.

## Introduction

Observational studies report that physical activity and metformin pharmacotherapy are associated with a 38% and 34% relative risk reduction in cancer‐specific mortality, respectively.^1^, ^2^ However, the biological mechanisms that mediate the associations between physical activity and metformin with clinical outcomes in cancer survivors remain incompletely understood.^3^, ^4^

Skeletal muscle secretes cytokines and other proteins, known as myokines, that exert paracrine and endocrine effects.^5^ Myokines regulate skeletal muscle hypertrophy, adipose tissue oxidation, insulin sensitivity and inflammation.^6^ Myokines, such as osteopontin and irisin, are associated with cancer development and progression in humans,^7^, ^8^ and preclinical models implicate myokines, such as oncostatin M and interleukin‐6 (IL‐6), in cancer cell growth, migration, invasion, apoptosis and angiogenesis.^9^, ^10^

Exercise alters myokine concentrations via the contraction of skeletal muscle fibres. Studies of exercise have been in animal models or healthy younger volunteers, examined the effects of an acute bout of aerobic or resistance activity or not utilized randomized comparisons.^11^ Metformin alters myokine concentrations, such as irisin, by increasing adenosine monophosphate‐activated protein kinase (AMPK) activity in skeletal muscle, sensitizing insulin‐resistant skeletal muscle and promoting muscle protein synthesis.^12^, ^13^ Collectively, these data suggest that exercise and metformin may have independent effects on myokine concentrations and, when paired, could produce synergistic effects consistent with an improved cancer prognosis.

Using archived biospecimens from a multi‐centre 2 × 2 factorial randomized design,^14^, ^15^ this analysis examined changes in the concentrations of 14 myokines after randomization to 12 weeks of exercise or metformin in breast and colorectal cancer survivors. The quantification of myokines was not pre‐specified in the study protocol; therefore, this analysis is exploratory and hypothesis generating.

## Methods

### Study design

This analysis used archived biospecimens from a phase II, multi‐centre, 2 × 2 factorial randomized trial designed to examine the effects of exercise, metformin or both interventions compared with control on biologic endpoints in breast and colorectal cancer survivors. Detailed study methods and the primary and key secondary endpoints have been reported.^14^, ^15^ Study centres included the Dana‐Farber Cancer Institute (Boston, MA, USA), Duke University (Durham, NC, USA) and Yale University (New Haven, CT, USA). Dana‐Farber Cancer Institute served as the coordinating centre. The Institutional Review Board for each centre approved the study. All participants provided written informed consent. The trial was registered on ClinicalTrials.gov as NCT01340300.

### Participants and study centres

Patients were eligible if they were diagnosed with stage I–III breast or colorectal cancer; completed surgical resection and any post‐operative systemic or radiation therapies (continued use of oral endocrine therapies and/or trastuzumab for breast cancer was allowed); self‐reported ≤120 min/week of moderate‐ to vigorous‐intensity physical activity; were age ≥ 18 years; had an Eastern Cooperative Oncology Group performance status of 0 (fully active) or 1 (ambulatory, but restricted in vigorous activity); had a non‐fasting plasma glucose concentration < 160 mg/dL or a fasting plasma glucose concentration < 126 mg/dL; obtained written physician approval; and provided written informed consent.

Patients were ineligible if they had another active primary cancer; had evidence of metastatic cancer determined by tumour marker concentrations or radiologic imaging; were using any pharmacotherapy for type 2 diabetes mellitus; had an absolute contraindication to participation in exercise; had a condition associated with an increased risk of metformin‐associated lactic acidosis; had known hypersensitivity or intolerance to metformin; or had any condition that, in the opinion of the investigator, made the subject unsuitable for participation.

### Randomization and blinding

Participants were stratified by body mass index (BMI; <30 vs. ≥30 kg/m^2^), sex (male vs. female) and cancer site (breast vs. colorectal) and randomized using a computer‐generated permuted block design with fixed block sizes. Randomization groups included 12 weeks of (1) exercise only; (2) metformin only; (3) exercise and metformin; or (4) control. Participants were not blinded to treatment assignments. Outcome measures were obtained by assessors blinded to treatment assignment.

### Exercise treatment plan

Participants randomized to exercise engaged in aerobic training for 12 weeks using a combination of twice‐weekly supervised activity with an exercise physiologist, supplemented by unsupervised home‐based activity. All exercise sessions began with a 5‐min warm‐up, followed by 30–60 min of moderate‐intensity exercise and concluded with a 5‐min cooldown. The primary exercise modality was walking, but cycling or other aerobic activities were permitted. The exercise physiologist utilized a heart rate monitor to educate participants about a level of exertion consistent with moderate intensity (e.g., 50–70% of the age‐predicted maximum heart rate). After aerobic training, 5–10 min of static stretching were performed. Under the guidance of the exercise physiologist, participants progressively titrated exercise volume by 10–30 min each week until the target of 220 min/week was achieved. After full titration, exercise volume was maintained at 220 min weekly until study completion.

### Metformin treatment plan

Participants randomized to metformin completed an initial dose‐titration interval of 2 weeks at 850 mg once per day. After 2 weeks, participants were evaluated by a physician, and laboratory measures were obtained to assess renal and liver function and glucose. Those who tolerated the initial metformin dose were titrated to 850 mg twice daily. Participants who experienced adverse events (e.g., gastrointestinal distress) were permitted to continue at 850 mg once per day for an additional week and then were re‐challenged at 850 mg twice per day after discussion with the investigative team. If dose titration was not tolerated after re‐challenge, the participant remained on 850 mg once per day until study completion.

### Myokine outcomes

All participants underwent a fasting (≥12 h) blood draw at baseline and 12 weeks. Ethylenediaminetetraacetic acid (EDTA)‐preserved plasma was stored at −80°C. Fourteen myokines, including apelin, fractalkine, brain‐derived neurotrophic factor (BDNF), osteonectin (SPARC), leukaemia inhibitory factor (LIF), interleukin‐15 (IL‐15), myostatin (GDF8), fatty acid binding protein 3 (FABP3), irisin, follistatin‐related protein 1 (FSTL‐1), oncostatin M, IL‐6, fibroblast growth factor 21 (FGF‐21) and osteocrin/musclin, were quantified using a Milliplex human myokine magnetic bead panel (Millipore Sigma, Burlington, MA, USA). Plasma (vs. serum) was used because plasma is more sensitive to detecting low‐abundance myokines.^16^ Baseline and 12‐week samples were assayed simultaneously and in duplicate. The intra‐ and inter‐assay precision was <10% and <15% for all analytes, respectively.

### Other measures

Demographic characteristics, including age, sex and race, were self‐reported. The smoking history was self‐reported. Cancer characteristics, including type, stage and treatment, were abstracted from the electronic medical record.

### Statistical analysis

The measurement of myokines was not pre‐specified in the study protocol; therefore, this analysis is exploratory and hypothesis generating. This study was powered to detect changes in the primary study endpoint of fasting plasma insulin.^14^ The sample size provided sufficient statistical power to identify effect sizes ≥ 0.4. There was no evidence of synergy between exercise and metformin for the reported myokine endpoints (results not shown); the inferential analysis compared the factorial main effects of the two interventions: metformin (with or without exercise) versus no metformin (with or without exercise) and exercise (with or without metformin) versus no exercise (with or without metformin). These represent the most efficient statistical contrasts in a randomized factorial trial.

Myokine concentrations were log‐transformed to approximate Gaussian distributions before regression modelling. Changes in myokine concentrations were estimated from baseline to 12 weeks in an intention‐to‐treat analysis using repeated‐measured mixed‐effects regression models. This statistical approach includes all available biomarker data and accounts for the correlation between repeated measures. All regression models included subject‐specific intercepts with fixed effects for time, treatment and time‐by‐treatment interactions. Models were adjusted for the baseline myokine concentration, randomization stratification factors and study centre to improve the precision of the estimated treatment effects. Treatment effects are presented as log‐transformed means and 95% confidence intervals (CIs). For each of the two main effects contrasts, we applied the sequential testing procedure of Holm to preserve the family‐wise alpha error of 5% across all myokine endpoints.^17^ Exploratory correlational analyses were conducted to determine the degree to which changes in energy‐balance‐related factors (detailed methods described elsewhere^14^) in all randomized groups were associated with changes in myokine concentrations. Stata Version 15.1 (Stata Corp., College Station, TX, USA) was used for all analyses.

## Results

Between September 2011 and December 2015, 139 participants were recruited and randomized, with data collection ending in April 2016. In this exploratory hypothesis‐generating analysis, 116 participants had sufficient archived plasma to analyse myokine concentrations (*Figure* *S1*). Participants excluded from this analysis did not differ from the overall study sample for demographic and clinical characteristics (results not shown).

### Baseline characteristics

The baseline characteristics of the study sample were similar between the randomized main effects groups (*Table* *1*). Age ranged from 33 to 79 years. BMI ranged from 17.3 to 45.0 kg/m^2^; 39% of participants were overweight (BMI 25.0–29.9 kg/m^2^), and 45% had obesity (BMI ≥ 30 kg/m^2^). Approximately two thirds of participants had breast cancer (64%), and one third had colorectal cancer (36%). The cancer stage was evenly represented with 37% stage I, 28% stage II and 35% stage III disease at diagnosis; the median [interquartile range, IQR] time since diagnosis was 1.9 years [1.0, 3.9], and the time since completing cancer treatment was 1.6 years [0.7, 3.8].

**Table 1:** Baseline characteristics of factorial groups at randomization (*N* = 116)

Characteristic	Factorial group, mean (SD) or *n* (%)			
	Exercise (*n* = 63)	No exercise (*n* = 53)	Metformin (*n* = 59)	No metformin (*n* = 57)
Age, years	55.3 (10.4)	55.4 (9.9)	54.5 (9.5)	56.2 (10.9)
Sex, %				
Male	11 (17%)	9 (17%)	11 (19%)	9 (16%)
Female	52 (83%)	44 (83%)	48 (81%)	48 (84%)
Race, %				
White	51 (81%)	44 (83%)	47 (80%)	48 (84%)
Black	4 (6%)	6 (11%)	5 (9%)	5 (9%)
Other	8 (13%)	3 (6%)	9 (11%)	4 (7%)
Body mass index, %				
<30 kg/m^2^	36 (57%)	27 (51%)	34 (58%)	29 (51%)
≥30 kg/m^2^	27 (43%)	26 (49%)	25 (42%)	28 (49%)
Type of cancer, %				
Colorectal	23 (37%)	19 (36%)	19 (32%)	23 (40%)
Breast	40 (63%)	34 (64%)	40 (68%)	34 (60%)
Time since diagnosis, years	3.3 (3.7)	3.2 (3.1)	3.4 (3.1)	3.1 (3.8)
Cancer stage, %				
I	25 (40%)	17 (32%)	26 (44%)	16 (28%)
II	14 (22%)	18 (34%)	13 (22%)	19 (33%)
III	23 (36%)	17 (32%)	20 (34%)	20 (35%)
Missing	1 (2%)	1 (2%)	0 (0%)	2 (4%)
Chemotherapy, %	41 (65%)	37 (70%)	40 (67%)	38 (67%)
Radiation, %	31 (49%)	25 (47%)	30 (51%)	26 (46%)

### Intervention compliance

Among participants randomized to exercise, 77% and 17% completed ≥50% and ≥90% of their initially prescribed exercise volume, respectively. Among participants randomized to metformin, 67% and 31% consumed ≥50% and ≥90% of their initially prescribed metformin dose, respectively. At 12 weeks, 91 (65%) participants completed their assigned intervention; reasons for premature discontinuation have been described.^14^ Participants who did not complete the study were more likely to be of White race (multivariable‐adjusted odds ratio: 3.59 [95% CI: 1.14, 11.36]); no other measured factors, including randomized group assignment and baseline concentrations of myokines, were associated with study completion.^15^

### Effects of exercise and metformin on myokine concentrations

**Figure 1:**
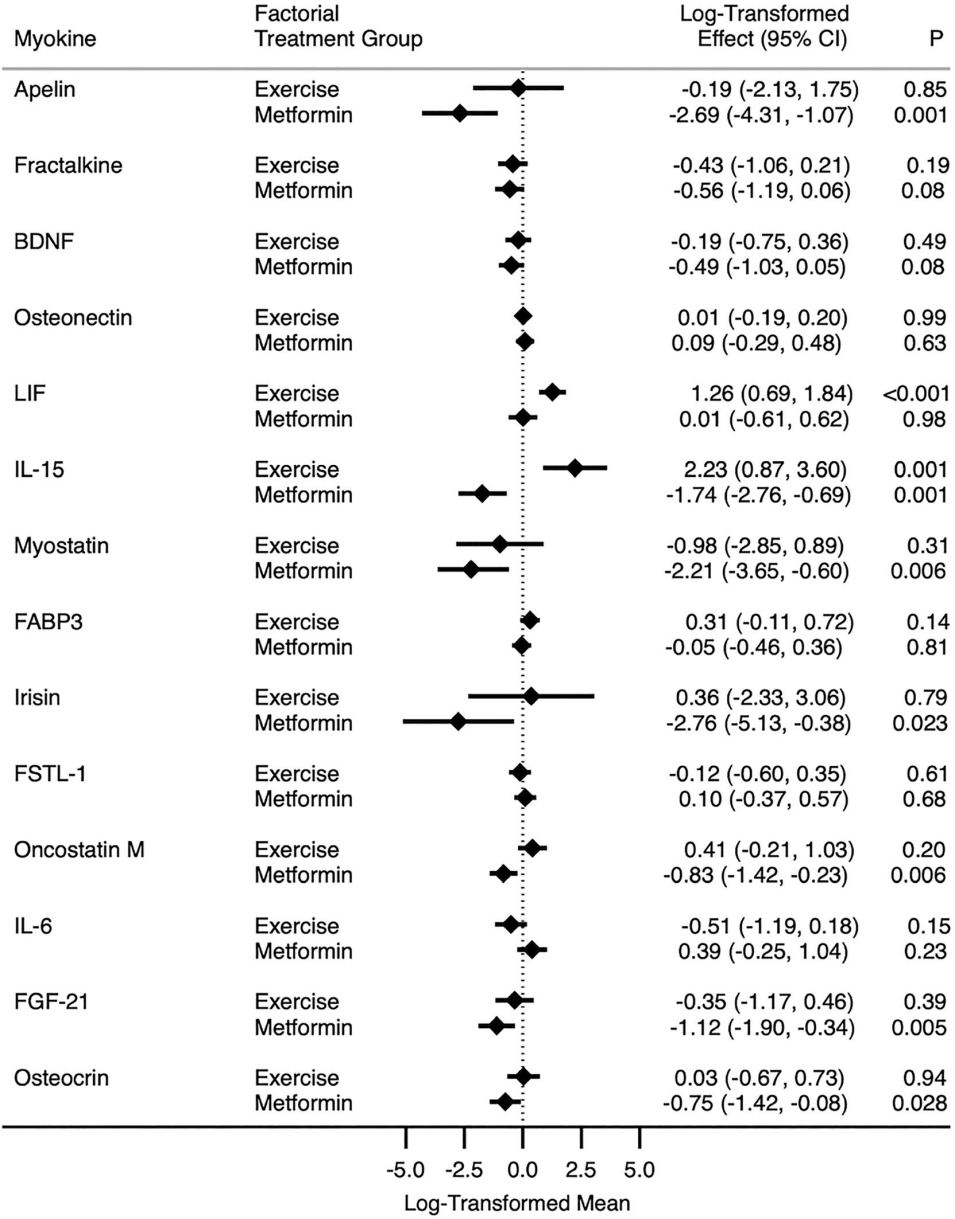
Mean myokine concentrations by exercise and metformin factorial groups. All myokines are in picograms per millilitre except osteonectin (nanograms per millilitre). Models adjusted for the baseline value of the dependent variable, body mass index (<30 vs. ≥30 kg/m^2^), gender (male vs. female), cancer site (colorectal vs. breast) and study centre (Dana‐Farber Cancer Institute vs. Duke University vs. Yale University). Factorial treatment group contrasts exercise (exercise group and exercise + metformin group) versus no exercise (metformin group and control group) and metformin (metformin group + metformin group and exercise group) versus no metformin (exercise group and control group). Nominal *P* values (unadjusted for multiple comparisons) are presented; see the text for adjusted *P* values. BDNF, brain‐derived neurotrophic factor; CI, confidence interval; FABP3, fatty acid binding protein 3; FGF‐21, fibroblast growth factor 21; FSTL‐1, follistatin‐related protein 1; IL‐6, interleukin‐6; IL‐15, interleukin‐15; LIF, leukaemia inhibitory factor.

At baseline, myokine concentrations were similar between the randomized main effects groups. By main effects analysis (e.g., contrasting the margins of the 2 × 2 table), randomization to exercise increased LIF (1.26 pg/mL, 95% CI: 0.69, 1.84; adjusted *P* = 0.001) and IL‐15 (2.23 pg/mL, 95% CI: 0.87, 3.60; adjusted *P* = 0.013) compared with randomization to no exercise. Randomization to metformin reduced apelin (−2.69 pg/mL, 95% CI: −4.31, −1.07; adjusted *P* = 0.014) and IL‐15 (−1.74 pg/mL, 95% CI: −2.79, −0.69; adjusted *P* = 0.013) compared with randomization to no metformin. Metformin decreased myostatin, irisin, oncostatin M, FGF‐21 and osteocrin; however, these changes were not statistically significant after correction for multiple comparisons (*Figure* *1* includes nominal [unadjusted] *P* values).

### Exploratory correlational analyses

We conducted additional hypothesis‐generating correlational analyses that consolidated all randomized groups to determine how changes in various energy‐balance‐related factors (e.g., anthropometric measures, physical activity, physical function and biomarkers of insulin metabolism) relate to changes in myokine concentrations (*Table* *S1*).

## Discussion

Among breast and colorectal cancer survivors, randomization to 12 weeks of aerobic exercise increased LIF and IL‐15, whereas randomization to 12 weeks of metformin reduced apelin and IL‐15. This is the first randomized study to explore the effects of exercise training and metformin on myokine concentrations in cancer survivors. These exploratory findings support the hypothesis that the relationships between physical activity and metformin and cancer outcomes may be mediated, in part, by changes in myokine concentrations. These data add to the growing literature implicating skeletal muscle myokines as key mediators in health and disease.^18^

IL‐15 concentrations increased with exercise. IL‐15 concentrations are positively associated with physical activity in healthy older adults.^19^ IL‐15 has been implicated in muscle‐adipose tissue cross‐talk.^20^ IL‐15 is negatively correlated with visceral adiposity in humans.^21^ In obese mice, the infusion of IL‐15 reduced adipose tissue by 33% but did not change skeletal muscle mass or protein content.^22^ Adiposity is a risk factor for breast and colorectal cancer incidence, recurrence and survival.^23^ Aerobic exercise reduces visceral adiposity in cancer survivors.^24^ If causal, exercise may influence disease recurrence or progression in cancer survivors, partly through IL‐15‐mediated reductions in adiposity.

Conversely, IL‐15 concentrations decreased with metformin. IL‐15 stimulates the proliferation of T cells, cytotoxic lymphocytes and natural killer (NK) cells.^25^ In Rhesus macaques, infusion of IL‐15 produced a 100‐fold increase in CD8^+^ T cells and a 7‐fold increase in NK cells.^26^ A phase I clinical trial of recombinant human IL‐15 infusion evaluated in patients with metastatic melanoma and renal cell cancer demonstrated significant changes in NK, γδ and CD8 memory T cells.^27^ IL‐15 is required for NK cell maturation and function.^28^ If causal, metformin may influence disease recurrence or progression in cancer survivors, partly through IL‐15‐mediated immune upregulation.

Exercise increased LIF. LIF induces the stimulation of platelet formation, proliferation of haematopoietic cells, osteoblast formation, neural formation and muscle satellite cell proliferation.^29^ An acute bout of aerobic or resistance exercise increases LIF mRNA expression in skeletal muscle; however, this change in LIF was not observed in plasma.^30^ Blocking LIF in a mouse model of colon carcinoma prevented the initiation and progression of cancer cachexia (e.g., muscle wasting) via the Janus kinase 2–signal transducer and activator of transcription 3 (JAK2–STAT3) pathway.^31^ This may partly explain the anabolic effects of exercise to prevent muscle wasting in cancer patients.^32^, ^33^

Metformin decreased apelin. Apelin may influence glucose metabolism.^34^ In obese mice, treatment with apelin increases total body energy expenditure and reduces insulin resistance by increasing glucose uptake in skeletal muscle.^35^ In patients with type 2 diabetes, metformin increases apelin concentrations.^36^ Insulin resistance is associated with an increased risk of cancer recurrence and death.^37^ Cancer cells have insulin receptors, and patients with hyperinsulinaemia and elevated concentrations of insulin‐like growth factors are significantly more likely to experience poor clinical outcomes.^38^

There are limitations to this trial. The measurement of myokines was not pre‐specified in the study protocol; therefore, this analysis is exploratory and hypothesis generating. We quantified 14 myokines hypothesized to mediate the association between physical activity and metformin with clinical outcomes in cancer survivors. Only three myokines were statistically significantly associated with exercise or metformin after adjustment for multiple hypothesis testing. It is uncertain if our study was statistically underpowered to detect small but potentially relevant treatment effects (e.g., effect sizes < 0.4) or if exercise or metformin does not affect these myokines. Our hypothesis‐generating analysis must be replicated. Replication could be achieved using stored biospecimens from previously completed trials or prospectively examined in future trials.

The timing of the 12‐week blood draw relative to the last bout of exercise or consumption of metformin was not standardized and ranged from 24 to 72 h. Myokines are dynamic, and the timing of the blood draw in this study may have influenced our findings. This limits our ability to attribute an acute versus chronic response to the interventions. We cannot determine if the myokines came from the skeletal muscle tissue or were produced from other sources. For example, IL‐15 is expressed by skeletal muscle, fibroblasts, astrocytes and epithelial cells.^39^ Participants were not recruited based on having an unfavourable myokine profile at baseline. It is unknown if the observed changes would be comparable in magnitude in a sample with an unfavourable myokine profile at baseline. All participants were prescribed the same dose of aerobic exercise and metformin; therefore, we cannot comment on any dose–response relationships or the effects of other exercise modalities, such as resistance training. We did not collect information on medical conditions that may influence metabolism and the paracrine and endocrine effects of myokines. Aside from anthropometric measures, we did not measure body composition; the availability of direct measures of fat and lean/muscle mass will be critical in future studies.

There are strengths in this trial. Using two distinct interventions allowed us to efficiently examine the effects of exercise and metformin on a shared set of biological endpoints. Although our multiplex panel was exploratory, we believe these data can inform the design of the next generation of targeted intervention studies. Future studies may wish to interrogate pre‐specified myokines and describe their acute and chronic responses to interventions with biological specimens (e.g., blood and tissue) that are systematically obtained before, during and after intervention.

This exploratory analysis of a randomized controlled trial demonstrates that exercise and metformin may elicit unique effects on myokine concentrations in breast and colorectal cancer survivors. The findings from this study support the hypothesis that the associations between physical activity and metformin with clinical outcomes in cancer survivors may be partly mediated by changes in myokine concentrations. These hypothesis‐generating observations warrant further basic, translational and clinical investigation and replication.

## Conflict of interest statement

The authors declare no relevant conflicts of interest. The results of the present study do not constitute endorsement by the American College of Sports Medicine (ACSM). The study results are presented clearly, honestly and without fabrication, falsification or inappropriate data manipulation.

## Supporting information


**Figure S1.** The flow of study participants.
**Table S1.** Relationship between change in energy‐balance‐related measures and change in log‐transformed (geometric mean) myokine concentrations during three months.
