# Supplementary material for: Effects of exercise or metformin on myokine concentrations in patients with breast and colorectal cancer: A phase II multi‐centre factorial randomized trial
Source: J Cachexia Sarcopenia Muscle. 2024 Jun 18;15(4):1520–7. doi: 10.1002/jcsm.13509 (PMC11294014; doi:10.1002/jcsm.13509)
Supplement: Supplementary file 1 — Figure S1. The flow of study participants. Table S1. Relationship between change in energy‐balance‐related measures and change in log‐transformed (geometric mean) myokine concentrations during three months. [file JCSM-15-1520-s001.docx]

**Supplementary Figure 1.** The flow of study participants


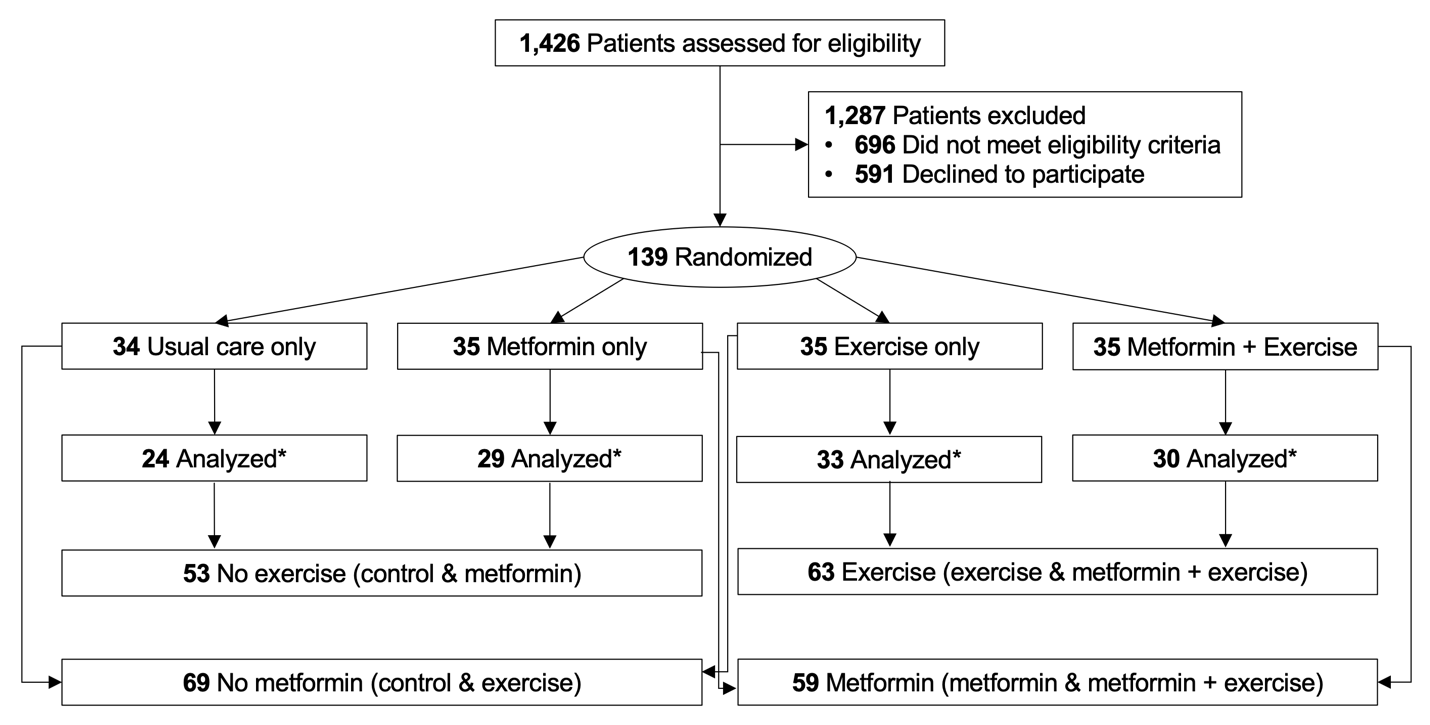


*Analyzed patients had an available plasma biospecimen for myokine assay.

**Supplementary Table 1.** Relationship between change in energy-balance-related measures and change in log-transformed (geometric mean) myokine concentrations during three months

| **Myokine** | **β (group-by-time interaction)** | | | | | | | | | | |
| --- | --- | --- | --- | --- | --- | --- | --- | --- | --- | --- | --- |
|  | **Body Mass** | **Waist Circ.** | **Physical Activity** | **6-Minue Walk** | **Insulin** | **Glucose** | **HOMA-IR** | **IGF-1** | **IGFBP-1** | **IGFBP-3** | **Leptin** |
| Apelin | −0.08** | — | — | −0.03*** | — | — | — | — | 0.80*** | 0.003*** | — |
| Fractalkine | — | — | — | −0.005** | — | — | — | — | — | — | — |
| BNDF | — | — | — | −0.004* | — | — | — | — | — | — | — |
| Osteonectin | — | — | — | — | — | — | — | — | — | — | — |
| LIF | — | — | — | −0.004* | — | −0.84*** | −0.24** | — | — | — | −0.03*** |
| IL-15 | −0.10*** | −0.06* | 0.005* | −0.01*** | — | −1.49*** | −0.50*** | −0.03*** | 0.34** | — | — |
| Myostatin | — | — | — | −0.01*** | — | — | — | — | −1.46* | — | 0.09* |
| FABP3 | — | — | — | — | — | — | — | — | — | — | −0.01* |
| Irisin | — | — | — | −0.03*** | — | — | — | −0.05** | 1.03*** | — | — |
| FSTL-1 | — | — | — | — | — | — | — | — | — | — | — |
| Oncostatin M | — | — | — | — | — | — | — | — | — | — | — |
| IL-6 | — | — | −0.004* | — | −0.01** | — | 0.24* | — | — | 0.001* | — |
| FGF-21 | — | — | — | −0.005* | — | — | — | — | — | — | — |
| Osteocrin | — | — | — | −0.008*** | — | — | — | -0.01* | — | — | — |

BNDF, Brain-Derived Neurotrophic Factor; LIF, Leukemia Inhibitory Factor; FABP3, Fatty Acid Binding Protein 3; FSTL-1, Follistatin-Related Protein 1; IL-6, Interleukin-6; FGF-21, Fibroblast Growth Factor 21. Models were adjusted for the baseline value of the dependent variable, body mass index (<30 kg/m^2^ vs ≥30 kg/m^2^), gender (male vs female), cancer site (colorectal vs breast), and study center (Dana Farber Cancer Institute vs Duke University vs Yale University). *P<0.05; **P<0.01; ***P<0.001.
